# Habitat Suitability Modelling for the Red Dwarf Honeybee (*Apis florea* (Linnaeus)) and Its Distribution Prediction Using Machine Learning and Cloud Computing

**DOI:** 10.1007/s13744-024-01220-y

**Published:** 2024-12-17

**Authors:** Shireen Ma’moun, Rasha Farag, Khaled Abutaleb, Amr Metwally, Abdelraouf Ali, Mona Yones

**Affiliations:** 1 Entomology Dept, Faculty of Science, Ain Shams Univ, Cairo, Egypt; 2 Bee Research Dept, Agricultural Research Centre, Plant Protection Research Institute, Ministry of Agricultural, Giza, Egypt; 3 National Authority for Remote Sensing and Space Sciences (NARSS), Cairo, Egypt

**Keywords:** Habitat suitability, Google Earth Engine, Satellite, Modelling, MODIS, *Apis florea*, Absence-only data

## Abstract

*Apis florea* bees were recently identified in Egypt, marking the second occurrence of this species on the African continent. The objective of this study was to track the distribution of *A. florea* in Egypt and evaluate its potential for invasive behaviour. Field surveys were conducted over a 2-year period, resulting in the collection of data on the spatial distribution of the red dwarf honeybees. A comprehensive analysis was performed utilizing long-term monthly temperature and rainfall data to generate spatially interpolated climate surfaces with a 1-km resolution. Vegetation variables derived from Terra MODIS were also incorporated. Furthermore, elevation data obtained from the Shuttle Radar Topography Mission were utilized to derive slope, aspect, and hillshade based on the digital elevation model. The collected data were subject to resampling for optimal data smoothing. Subsequently, a random forest model was applied, followed by an accuracy assessment to evaluate the classification output. The results indicated the selection of the mean temperature of coldest quarter (bio11), annual mean temperature (bio01), and minimum temperature of coldest month (bio06) as temperature-derived parameters are the most important parameters. Annual precipitation (bio12) and precipitation of wettest quarter (bio16) as precipitation parameters, and non-tree vegetation parameter as well as the elevation. The calculation of the Habitat Suitability Index revealed that the most suitable areas, covering a total of 200131.9 km^2^, were predominantly situated in the eastern and northern regions of Egypt, including the Nile Delta characterized by its fertile agricultural lands and the presence of the river Nile. In contrast, the western and southern parts exhibited low habitat suitability due to the absence of significant green vegetation and low relative humidity.

## Introduction

The red dwarf honeybee, *Apis florea* (Hymenoptera: Apidae), is known as the smallest bee species with a wide distribution in various regions. Originating in Asia, including countries like Pakistan, India, Sri Lanka, Thailand, Southeast China, and Malaysia, *A. florea* has naturally spread westward unintentionally, aided by global mobility (Mogga and Ruttner 1988; Hepburn et al. 2005 and Ghassemi-Khademi et al. 2022a, b a,b). In addition to its Asian origins, red dwarf honeybees have been found in the Arab Gulf nations of Oman, Iraq, and Saudi Arabia (Hepburn et al. 2005), as well as Jordan (Haddad et al. 2008), Yemen (Svvongsiri et al. 1996), and Sudan (Mogga and Ruttner 1988), bordering the Red Sea. *Apis florea* has more recently been reported in Eilat and Aqaba (Haddad et al. 2008; Moritz et al. 2010), and its presence has extended to Egypt, marking the second instance of this species on the African continent (Shebl 2017).

This successful colonization in the Arabian Peninsula and Africa demonstrates *A. florea*’s competitive ability, particularly against *Apis mellifera*, and its adaptation to hot environmental conditions in urban and rural areas (El-Shafie et al. 2002). Furthermore, the existence of *A. florea* in Sudan since 1985 has been documented (Mogga and Ruttner 1988; Hepburn et al. 2005). *Apis florea* serves significant ecosystem functions (Mogga and Ruttner 1988; Hepburn et al. 2005; Oldroyd and Nanork 2009) but make Anaphylactic shock and suddenly death as cause of allergies reaction of bee sting (Schwartz et al. 1986; Ollert and Blank 2015; Fakhar et al. 2022).

*Apis florea* constructs a single outdoor comb instead of residing in artificial hives. Nest structure varies depending on the environment, with a tiny comb no larger than a human hand. They can be found nesting in corners, on rock cliffs, trees, home gardens, buildings, rooftops, or among household items, often preferring shaded locations. *Apis florea* is highly migratory, preferring migration over defence when colonies are disturbed by predators or pests (Radjabi et al. 2018).

The increased presence of *A. florea* within *A. mellifera*’s range raises concerns. *Apis florea* produces significantly less honey, around 300–450g, limiting its utilization for honey production compared to *A. mellifera* (Mogga and Ruttner 1988; Hepburn et al. 2005). Their nesting habits in bushes and relatively low aggression levels contribute to their inconspicuousness to humans over extended periods (Oldroyd and Wongsiri 2009). *Apis florea* is a migratory species, traveling in swarms and following nectar flows (Akratanakul 1977). They have great reproductive potential and high mobility, essential characteristics for invasive species (Schouten et al. 2019). During foraging, *A. florea* competes well with *A. mellifera* (Koeniger 1976) and may even usurp *A. mellifera* colonies (Chahal et al. 1986; El-Niweiri et al. 2005). The introduction of diseases and pests through *A. florea* honeybees, including the parasite mite *Euvarroa sinhai*, is a significant concern (Akratanakul and Burgett 1976; Koeniger 1990). These diseases can have severe and unpredictable effects when contaminating *A. mellifera* colonies, similar to the impacts of *Varroa* destructor infestations. Native species are vulnerable to these diseases, while the invader remains relatively unharmed (Goulson 2003; Shavit et al. 2009; Aizen et al. 2020). Therefore, predicting the possible propagation of red dwarf bees in Africa and Europe is very crucial for adequate management plans especially under climatic changes.

In this study, we aim to assess the spread of *A. florea* in Egypt and evaluate its potential for invasion. Our study focuses on mapping the habitat suitability of *A. florea* in Egypt using existence data collected during the years 2020 and 2021. We incorporate various factors, including satellite-driven vegetation and bioclimatic parameters, and employ machine learning algorithms and cloud computing capabilities in Google Earth Engine and Google Colab to analyse the data. The findings of this research contribute to our understanding of the distribution patterns of *A. florea* and provide valuable insights into their habitat preferences and ecological requirements. By involving local residents in the data collection process, this study showcases the importance of community engagement and citizen science in academic research endeavours (Bickerman-Martens et al. 2017; Hall et al. 2021).

## Material and Methods

### Study Area

Egypt is a transcontinental nation that is part of both the Middle East and North Africa. Its official name is the Arab Republic of Egypt, with a vast territory spanning approximately 1,010,450 km^2^. Situated between latitude 26°50′8.76″N and longitude 30°47′44.37″E, Egypt is strategically positioned at the crossroads of Africa, Asia, and Europe (CIA World Factbook, 2021).

Geographically, Egypt is bordered by the Red Sea to the east and the Mediterranean Sea to the north, serving as vital maritime links to various international trade routes (World Bank, 2021). Libya forms its western border, while Sudan marks its southern boundary (CIA World Factbook, 2021). This geopolitical location has endowed Egypt with a significant role in regional politics and commerce, making it an important player in the Middle East and North Africa (Kausch 2015).

The climate of Egypt is predominantly arid, characterized by limited rainfall throughout the year. The majority of precipitation occurs along the coastal regions, with the Nile Delta experiencing slightly higher rainfall due to its proximity to the Mediterranean Sea (Roushdi 2022). Summers in Egypt are scorching and arid, spanning from May to September, with temperatures often surpassing 40°C in the inland areas. Winters, on the other hand, are relatively mild and chilly, particularly in the northern parts of the country (Kottek et al. 2006).

### Dataset Description

#### Presence Data

The geographical distribution of *A. florea* was systematically documented over a span of 2 years. Close collaboration was established with local residents all over the study area, who willingly participated and provided valuable inputs. Residents promptly reported the discovery of honeybee nests on their premises via telephone, acting as citizen scientists contributing to the research effort (Bickerman-Martens et al. 2017; Hall et al. 2021).

**Fig. 1:**
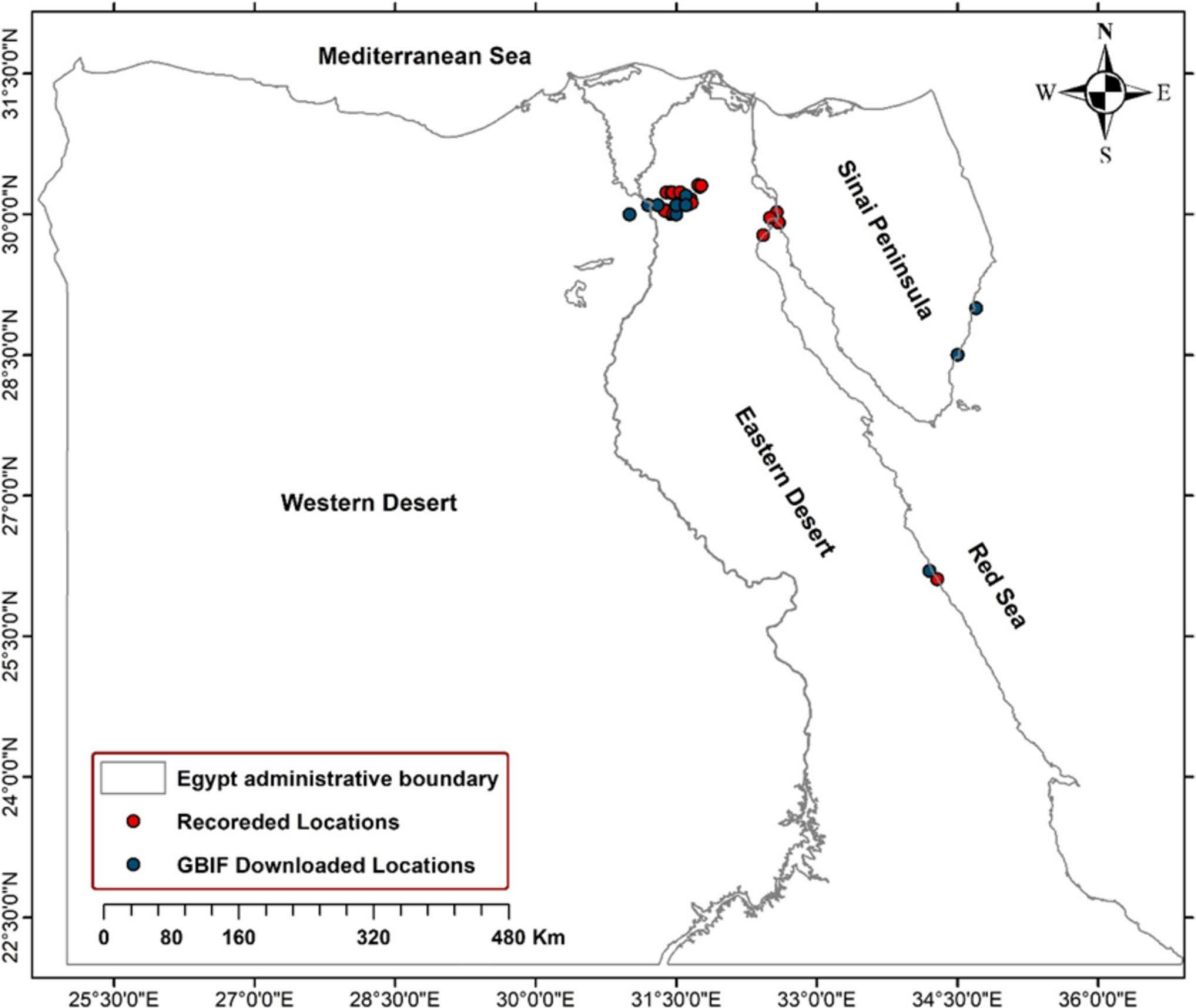
Study area and reported location of *Apis florea* in Egypt

To ensure comprehensive data collection, a designated team member conducted site visits to accurately record the precise coordinates and other relevant information of each honeybee nest. These on-site visits were essential for validating the reported sightings and confirming the presence of *A. florea* in the specified locations. A total of 33 nests were successfully collected from diverse locations across the study area, representing a significant sample size for further analysis and study. More over 23 data points of *A. florea* were obtained in the study area from the GBIF platform (https://gbif.org) as illustrated in Fig. 1.

#### Bioclimatic Data

**Fig. 2:**
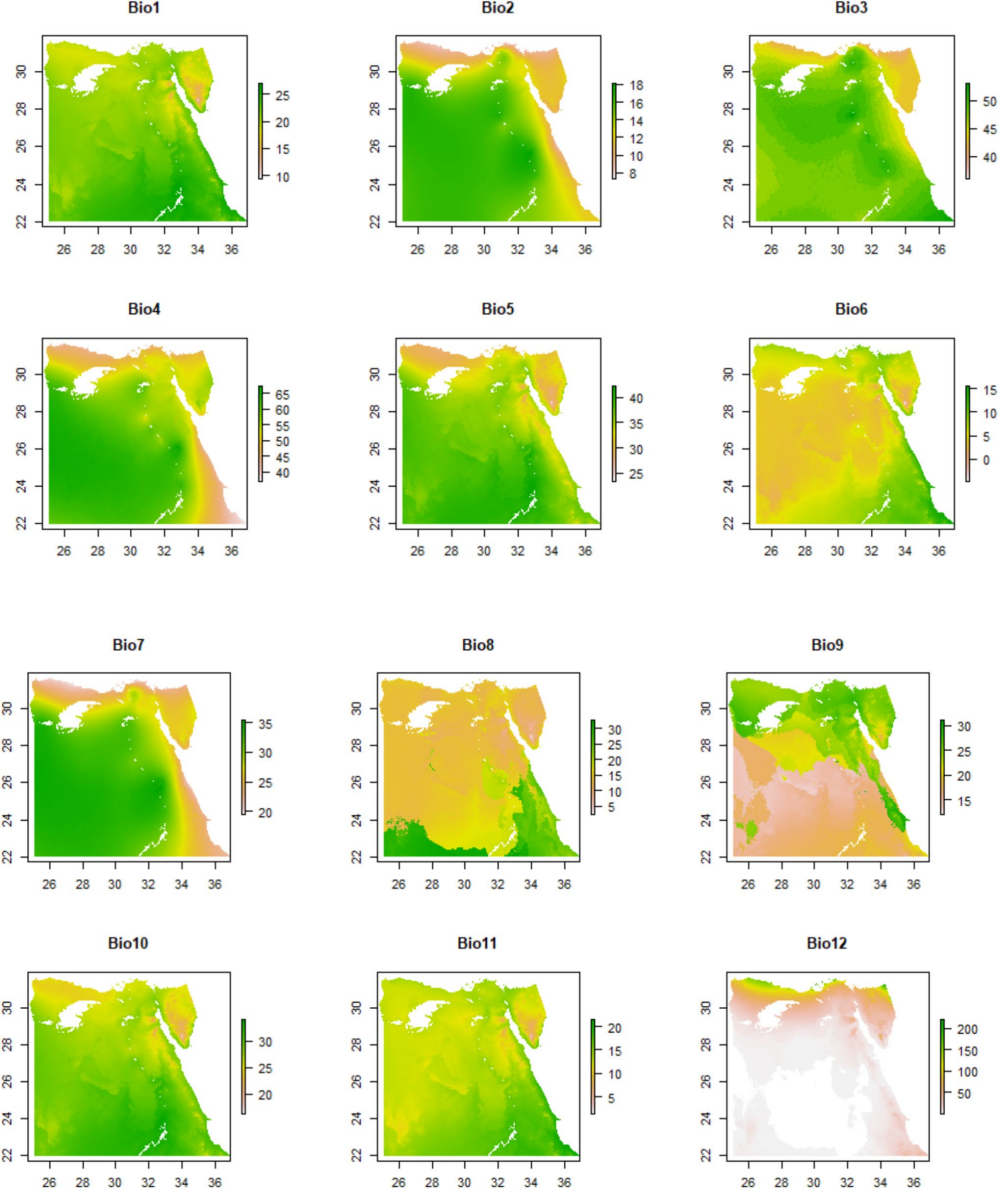

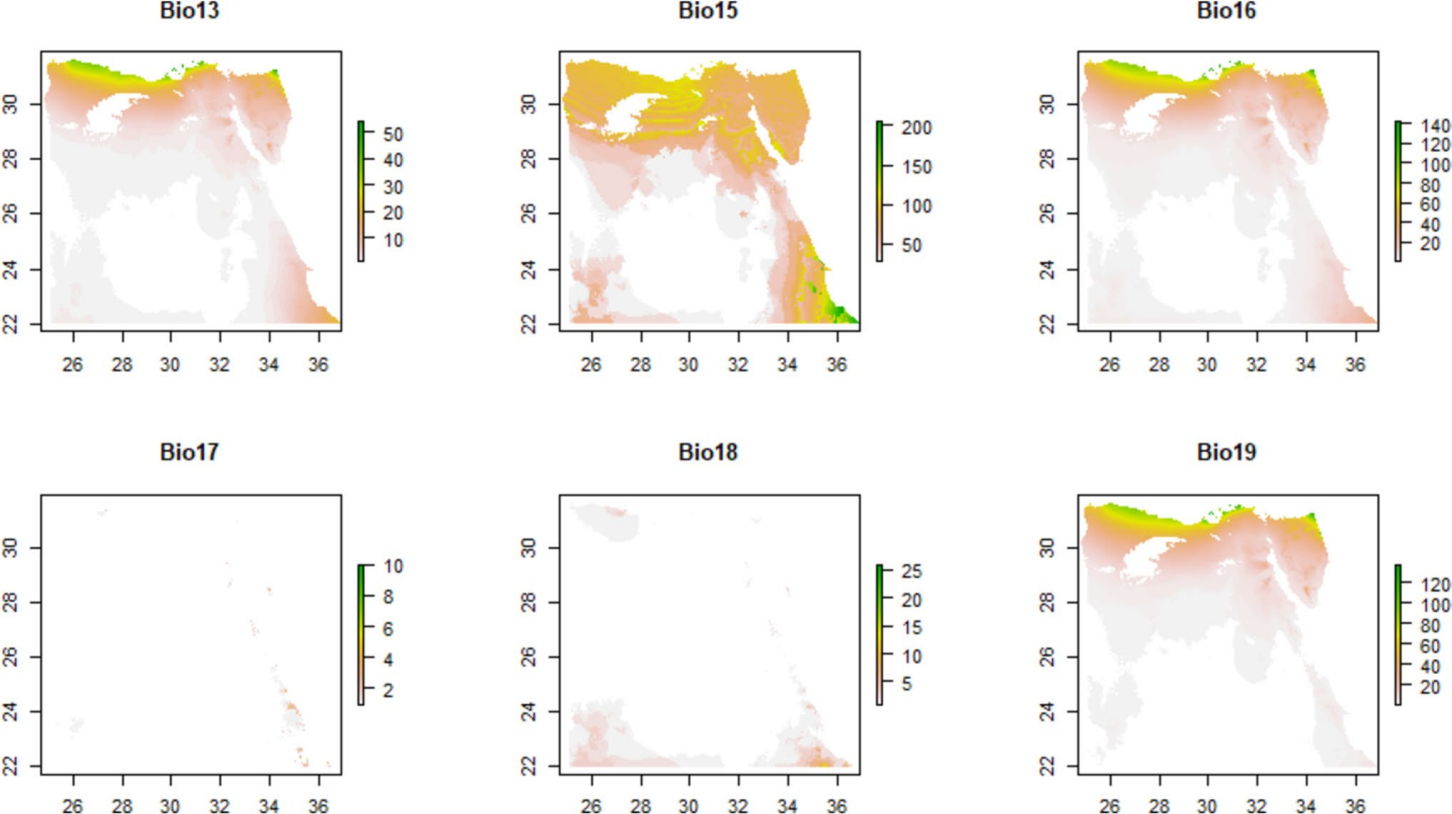
Bioclimatic parameter description and summary information

The bioclimatic data (https://www.worldclim.org/data/bioclim.html) presents and elucidates the diverse parameters that were examined and chosen for the mapping exercise. All of the listed parameters (Fig. 2) were derived from the monthly temperature and rainfall data to generate climate surfaces with a spatial resolution of 1 km. The time span covered the years 1950 to 2000 (Hijmans et al. 2005).

#### Vegetation Data

**Fig. 3:**
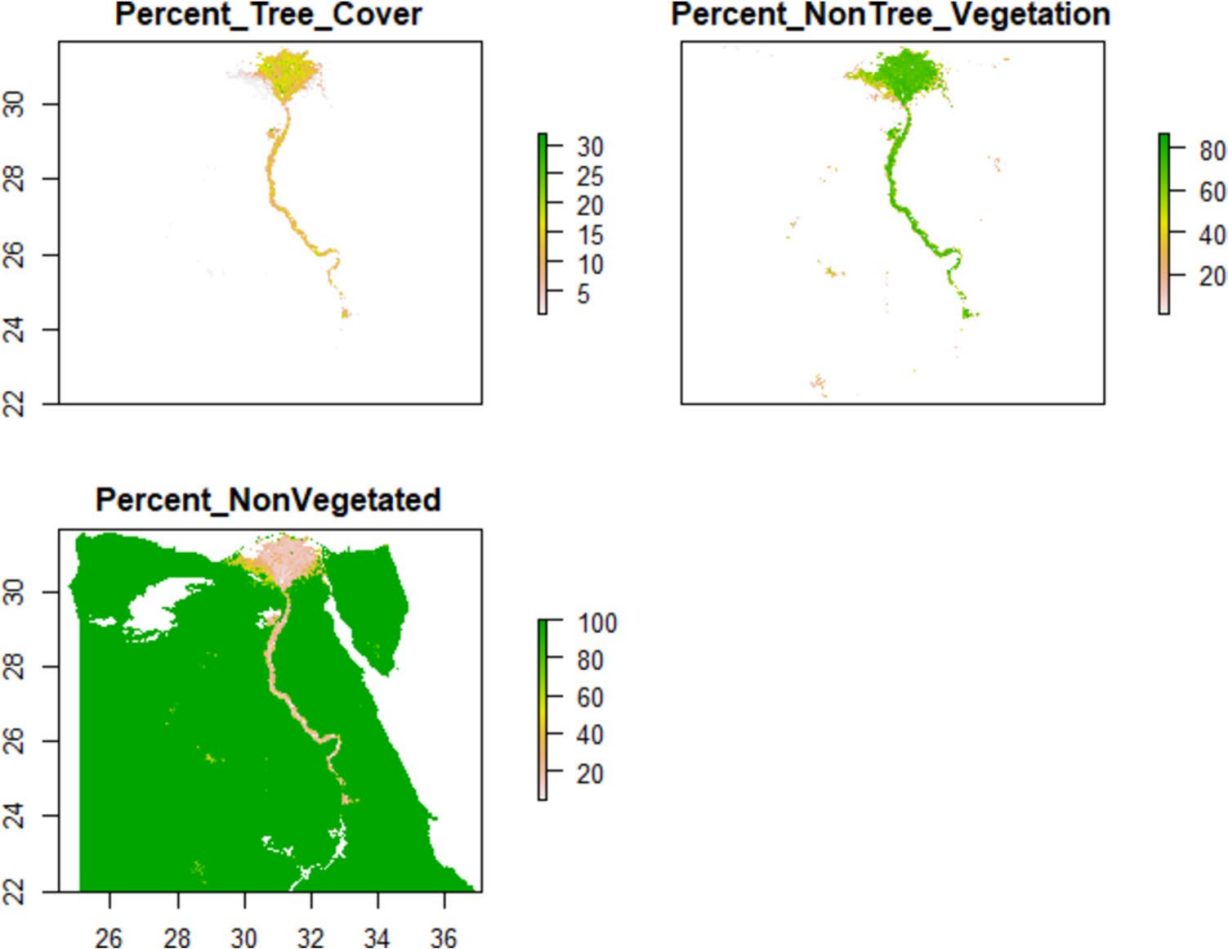
Vegetation parameters

The MODIS Terra Vegetation Continuous Fields (VCF) data product presented at https://lpdaac.usgs.gov/products/mod44bv006/ provides a summary of the vegetation variables utilized in this study. All the parameters correspond to yearly satellite data products derived from Terra MODIS (Moderate Resolution Imaging Spectroradiometer). The dataset spans from 2000 to 2021, encompassing a considerable temporal scope. Moreover, the spatial resolution employed for this analysis is set at 250 m, ensuring an appropriate level of detail for the study area (Fig. 3).

#### Topographic Data

**Fig. 4:**
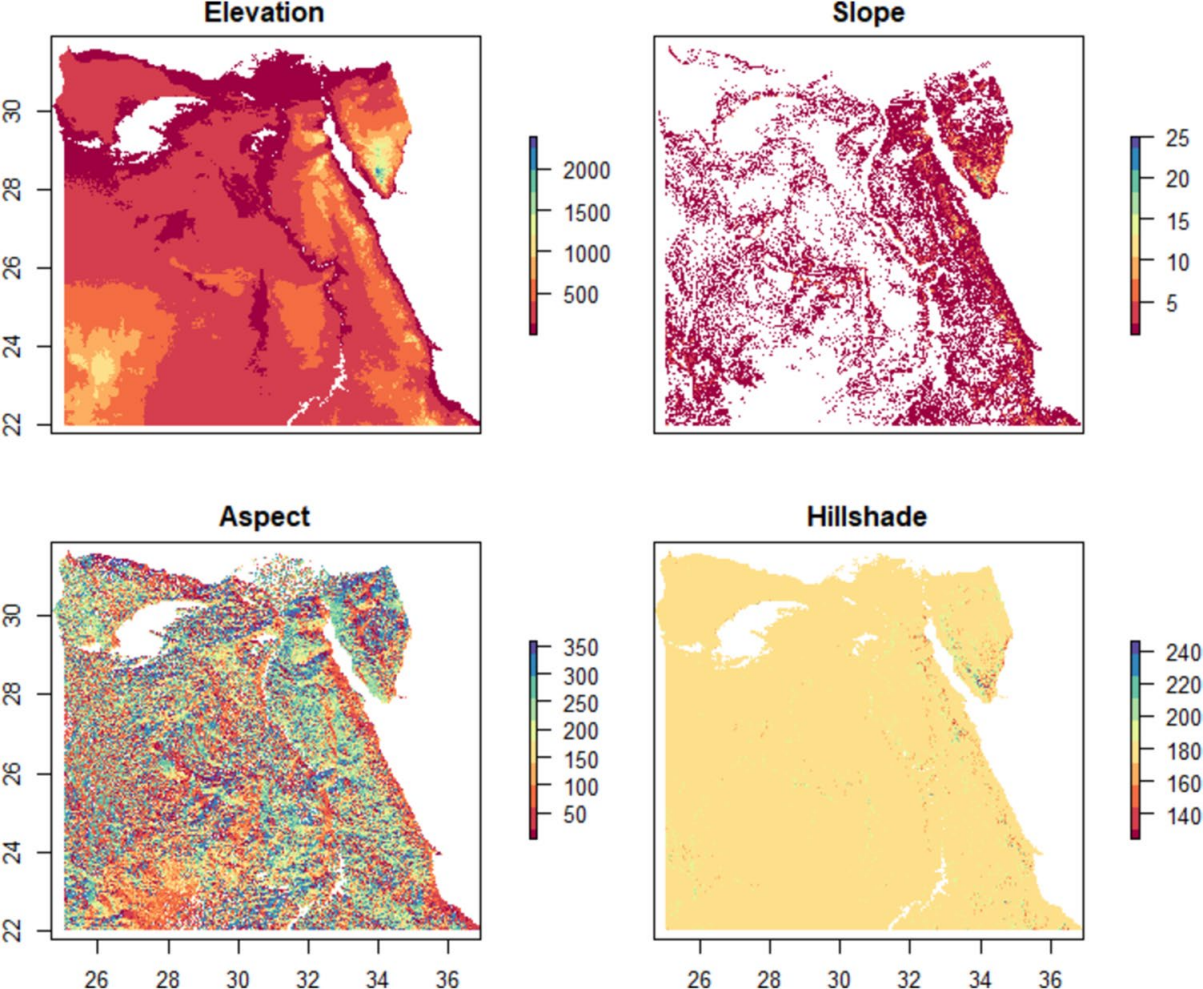
Elevation, slope, aspect, and hillshade from the terrain digital elevation model

Elevation data acquired from the Shuttle Radar Topography Mission (SRTM) were utilized to compute slope, aspect, and hillshade, employing a digital elevation model (DEM) with a spatial resolution of 30m (Fig. 4).

### Data Processing and Analysis

#### Data Resampling

In order to achieve uniformity in pixel size, data resampling was conducted to account for the disparate spatial resolutions across the various datasets (Mitas and Mitasova 1999; Mookambiga and Gomathi 2016). All data were aligned with the low-resolution dataset (bioclimatic). Raster resampling encompasses the adjustment of pixel size by means of either enlargement or reduction (Sillero and Barbosa 2021; Eyelade et al. 2022). The three predominant algorithms employed in raster resampling include nearest neighbour, bilinear interpolation, and cubic convolution (Mitas and Mitasova 1999). For this particular study, bilinear interpolation was implemented to augment the pixel size from different resolutions to 1km (Lahiri and Lahiri 2003). This method entails computing the weighted average of the four pixels in the original image that are nearest to the new pixel location. Through this averaging process, the original pixel values are modified, resulting in entirely new digital values in the output image (Usery et al. 2004; Afwani and Danoedoro 2019). Bilinear interpolation is generally recommended for continuous data, albeit it may introduce some degree of data smoothing (Mitas and Mitasova 1999; Lahiri and Lahiri 2003).

### Create Pseudo-Absences Spatial Data Blocks

Species distribution models (SDMs) are essential tools in ecological research, helping us understand species’ habitat preferences and predict their spatial distribution patterns (Mitas and Mitasova 1999). To create SDMs, we need data on species’ presence and the available environmental conditions, also known as pseudo-absence data (Elith et al. 2006). In this study, we utilized a widely used *k*-means clustering algorithm, which groups similar data points together based on Euclidean distance (Hartigan and Wong 1979). The clustering analysis allowed us to identify areas with similar environmental conditions and presence records, offering valuable insights into species’ ecological niches (Jiménez-Valverde et al. 2008).

For generating pseudo-absences, we randomly created points within dissimilar pixels relative to the presence data. This common approach in SDMs helps simulate absence data and prevents bias in model predictions. Incorporating random pseudo-absences enables the model to capture background environmental conditions more effectively, improving its ability to distinguish suitable habitats from unsuitable ones.

The study contributes to the existing SDM literature, emphasizing the significance of robust presence data and the use of appropriate pseudo-absences to enhance model accuracy. The *k*-means clustering method and random pseudo-absence generation provide valuable insights into species distribution patterns and ecological niche modeling. Ongoing research to refine these techniques will contribute to a better understanding of species-environment relationships and support conservation efforts.

#### Feature Importance and Parameter Selection

One of the key advantages of random forest is its ability to assess the importance of different features in making predictions. Feature importance provides insights into which features have the most influence on the model’s predictions. This can be particularly valuable for feature selection, model interpretation, and understanding the underlying data. Each decision tree in the random forest is split based on criteria that minimize impurity, such as Gini impurity or entropy. When assessing feature importance, random forest measures how much each feature decreases the impurity in the nodes where it is used. For each feature, the algorithm calculates the total reduction in impurity across all nodes where the feature is used for splitting. This reduction is then averaged across all trees in the forest.

Features that frequently result in large reductions in impurity are considered more important. Features with higher importance scores are more influential in predicting the target variable. These features contribute significantly to reducing uncertainty and improving the model’s predictive performance. Features with lower scores have a minimal impact on the model’s predictions. These features might be redundant or less relevant to the outcome. Feature importance was calculated as an average of 10 different runs of the trained models using 10 different seeds [11, 17, 22, 34, 54, 68, 96, 108, 224, 312] for model generalization.

#### Model Fitting, Validation, and Accuracy Assessment

Random forest (RF) is a popular nonparametric ensemble predictor used extensively in academic research. It relies on an ensemble of decision trees trained or bootstrapped with a subset of training samples (Breiman 2001; Pal 2005; Gislason et al. 2006). RF’s appeal lies in its ability to handle large, non-parametric categorical data, effectively manage outliers, and resist overfitting issues. The algorithm combines multiple decision trees using binary decisions, growing each tree based on random sampling with replacement from the original training data (Breiman 2001).

In RF, each decision tree contributes a single vote to assign the most frequent class to the input data. The final classification output is determined by majority vote, selecting the class with the highest number of votes out of the total (*N* votes) (Breiman 2001; Pal 2005; Gislason et al. 2006). Users have the flexibility to define the number of variables (mtry) and trees (ntree) based on specific research requirements (Pal 2005; Gislason et al. 2006). The presence and pseudo-absence data were extracted using Google Earth Engine (GEE) and subsequently downloaded for offline analysis in R software, where the random forest hyperparameter tuning was performed. Specifically, a total of 440 pseudo-absence data points were generated, and the corresponding pixel values for all biophysical and environmental parameters were extracted. This process was similarly conducted for the presence data points.

This section outlines the methodology employed to evaluate the performance of the random forest (RF) model for binary classification, focusing on the impact of varying hyperparameters using 5-fold cross-validation. The analysis was conducted in R, with particular attention given to the handling of class imbalance during data sampling. The random forest algorithm was evaluated across a range of hyperparameter settings:

- Number of trees (ntree): evaluated at 50, 100, 200, 500, 1000, and 1500 trees.
- Number of features considered at each split (mtry): assessed with values of 3, 5, 7, 9, 10, 15, and 20 features.

For each combination of ntree and mtry, a random forest model was trained on the training folds and validated on the corresponding validation fold. The dataset used for this study was imbalanced for binary classification tasks. To address potential class imbalance, the following sampling strategies were applied:

- Negative class: instances were randomly selected without replacement to ensure diversity and representativeness in the training data.
- Positive class: instances were sampled with replacement to maintain a sufficient number of examples and counteract any imbalance issues.

A 5-fold cross-validation approach was employed to assess the performance of the random forest model. The dataset was divided into 5 equal-sized folds. Each fold was used once as the validation set, while the remaining 4 folds were utilized for training. This process was repeated five times, ensuring that every data point was used for both training and validation exactly once. This cross-validation was repeated in a for loop for 10 times to make sure all the absence data were all used against the absence data. The top preforming set of parameters (ntree and mtry) were used in GEE for the RF model implementation. The out-of-bag error and validation accuracy were used as a model performance selection.

Accurate assessment of classification results is crucial for evaluating the reliability of image classification algorithms. Metrics such as user’s accuracy, producer’s accuracy, overall accuracy, and the Kappa coefficient are commonly used to quantitatively evaluate classification accuracy in tools like GEE (Lambin and Strahlers 1994; Congalton 1991). The overall accuracy, user’s accuracy, and producer’s accuracy can be calculated using the following equation:

$$\text{Overall Accuracy}= \frac{\text{Total number of pixels accurately classified}}{\text{Total number of referenced pixels}}\times 100$$

While the producer’s accuracy is a measure of the accuracy of the produced map as evaluated by the mapmaker (Mawasha and Britz 2022) and can be calculated using the following equation:

$$\text{Producer Accuracy}= \frac{\text{Total number of correct pixels in a category}}{\text{Total reference pixels }(\text{i}.\text{e}.,\text{ row total})} \times 100$$

The user’s accuracy refers to the perspective of the map user (Mawasha and Britz 2022) and can be calculated using the following equation:

$$\text{User Accuracy}= \frac{\text{Total number of correctly correct pixels in a category}}{\text{Total reference pixels }(\text{i}.\text{e}.,\text{ column total})} \times 100$$

Kappa values are classified into three possible ranges: a value greater than 80% is a strong agreement, a value between 40 and 80% is moderate agreement, and when a value less than 40% is poor agreement (Mawasha and Britz 2022). The Kappa index can be calculated using the following equation:

$$K= \frac{N {\sum }_{i=1}^{r}Xii-{\sum }_{i=1}^{r}({x}_{i+}* {x}_{+i})}{{N}^{2}-{\sum }_{i=1}^{r}({x}_{i+}* {x}_{+i})}$$

where $r$ is the number of rows, ${x}_{i}$ is the number of observations in row $i$ and column $i$, ${x}_{i+,}$ and ${x}_{+i}$ are the marginal totals of row and column, and $N$ is the total number of observed pixels.

## Results

Table 1 represents the summary statistics for various parameters used in modelling habitat suitability for *Apis florea* in Egypt. Each parameter provides important information about the environmental conditions that influence the suitability of habitats for these bees. The summary statistics include minimum, quartiles, median, mean, and maximum values for each parameter.

**Table 1:** Summary of statistics of all parameters over the study area

Name	Min	1^st^ Qu	Median	3^rd^ Qu	Max
Bio1	9.4	20.6	21.7	23.6	27
Bio2	7.3	13.4	16.1	16.7	18.1
Bio3	36	47	48	49	53
Bio4	37.13	54.16	60.36	63.73	67.65
Bio5	23.4	34.8	37.3	38.9	42.4
Bio6	− 4.7	3.9	5.1	7	15.7
Bio7	19.5	27.9	32.6	34	35.5
Bio8	2.4	13.8	14.6	18.2	32.6
Bio9	12	16.3	18.3	24.3	31.4
Bio10	16.4	27.3	28.9	30.3	34.2
Bio11	1.5	12.5	13.2	15.1	21.6
Bio12	1	2	6	24	227
Bio13	1	1	2	6	55
Bio14	1	1	1	1	1
Bio15	28	37	62	86	205
Bio16	1	2	4	15	145
Bio17	1	1	1	1	10
Bio18	1	1	1	2	26
Bio19	1	1	4	17	141
Elevation	1	167	264	412	2467
Slope	1	1	1	2	25
Aspect	1	76	180	270	359
Hillshade	108	180	180	181	255
Percent_Tree_Cover	1	3	11	15	38
Percent_NonTree_Vegetation	1	39	66	71	87
Percent_NonVegetated	4	100	100	100	100

The analysis of temperature variables (bio1 to bio11) indicates a moderate temperature range (9.4 to 27°C) with a median of 21.7°C, suggesting favourable conditions for *A. florea*. The diurnal temperature range (bio2) shows moderate variation (7.3 to 18.1°C) throughout the year, which is also beneficial for the species (Beaumont et al. 2005; Elith et al. 2006; Parichehreh et al. 2022). The isothermality (bio3) reveals a relatively stable temperature regime (range, 36 to 53), promoting habitat suitability. However, temperature seasonality (bio4) exhibits significant fluctuations (range, 37.13 to 67.65°C), which may influence the species’ habitat suitability. The maximum temperature of the warmest month (bio5) indicates relatively high temperatures (23.4 to 42.4°C), favouring *A. florea*. Conversely, the minimum temperature of the coldest month (bio6) suggests cold temperatures (− 4.7 to 15.7°C) during winter months, potentially impacting the species’ distribution and activity. The temperature annual range (bio7) reveals a moderate temperature range (19.5 to 35.5°C), supporting *A. florea* populations (Beaumont et al. 2005; Elith et al. 2006; Parichehreh et al. 2022). The mean temperature of the wettest quarter (bio8) exhibits a moderate range (2.4 to 32.6°C) during the wettest period, while the mean temperature of the driest quarter (bio9) indicates relatively high temperatures (12 to 31.4°C) during this time. The mean temperature of the warmest quarter (bio10) suggests high temperatures (16.4 to 34.2°C) during the warmest period, whereas the mean temperature of the coldest quarter (bio11) reflects moderate to low temperatures (1.5 to 21.6°C) during winter months.

These temperature patterns, as identified through various studies (Beaumont et al. 2005; Elith et al. 2006; Parichehreh et al. 2022), influence the availability of resources and habitat suitability for *A. florea*.

Moving on to precipitation, Bio12, the annual precipitation, suggested consistently low levels throughout the year, indicating an arid or semi-arid environment. Bio13, the precipitation of the wettest month, aligned with the arid conditions observed in Bio12, indicating low precipitation during the wettest month. Bio14 showed little variation in precipitation between seasons. Bio15 indicated relatively low precipitation during the driest period. Bio16 represented low to moderate rainfall during the wettest quarter. Bio17 indicated consistently low rainfall during the driest month. Bio18 and Bio19 suggested low precipitation during the warmest and coldest quarters, respectively (Beaumont et al. 2005; Elith et al. 2006; Parichehreh et al. 2022).

Moving on to precipitation, data illustrated in Table 1 indicated that the study area exhibits low levels of precipitation throughout the year, suggesting an arid or semi-arid environment. The annual precipitation (Bio12) ranges from 1 to 227mm, with a median of 6mm, while the precipitation of the wettest month (Bio13) ranges from 1 to 55mm, with a median of 2mm. These values confirm the scarcity of rainfall, even during the wettest period. Moreover, the parameter representing precipitation seasonality (Bio14) shows minimal variation between seasons, with a constant value of 1. The precipitation of the driest quarter (Bio15) ranges from 28 to 205mm, with a median of 62mm, indicating relatively low rainfall during the driest period. Similarly, the precipitation of the wettest quarter (Bio16) ranges from 1 to 145mm, suggesting low to moderate rainfall during the wettest period. The parameters representing precipitation of the driest month (Bio17), warmest quarter (Bio18), and coldest quarter (Bio19) all exhibit consistently low to moderate levels of rainfall, with values ranging from 1 to 26mm and 1 to 141mm, respectively. Overall, the analysis demonstrates the arid nature of the study area, characterized by limited and sporadic precipitation throughout the year.

Elevation plays a crucial role in determining suitable habitats for *A. florea*, as it ranges from 1 to 2467m above sea level. This diverse elevational range provides varied habitats that can support the species. The slope, ranging from 1 to 25°, represents the steepness of the terrain and can influence the distribution and movement of *A. florea*. Similarly, the aspect, ranging from 1 to 359°, indicates varied exposure to sunlight and wind, which in turn affects the microclimatic conditions.

The percentage of tree cover (Percent_Tree_Cover) ranges from 1 to 38, suggesting varying degrees of tree-covered areas that can provide suitable habitats for *A. florea*. Similarly, the percentage of non-tree vegetation (Percent_NonTree_Vegetation) ranges from 1 to 87, indicating different proportions of non-tree vegetation that can influence foraging resources for these bees. Conversely, the percentage of non-vegetated areas (Percent_NonVegetated) ranges from 4 to 100, highlighting barren or unvegetated areas that may limit the availability of suitable habitats (Wu et al. 2018).

### Random Forest Model Hyperparameter Tuning

**Fig. 5:**
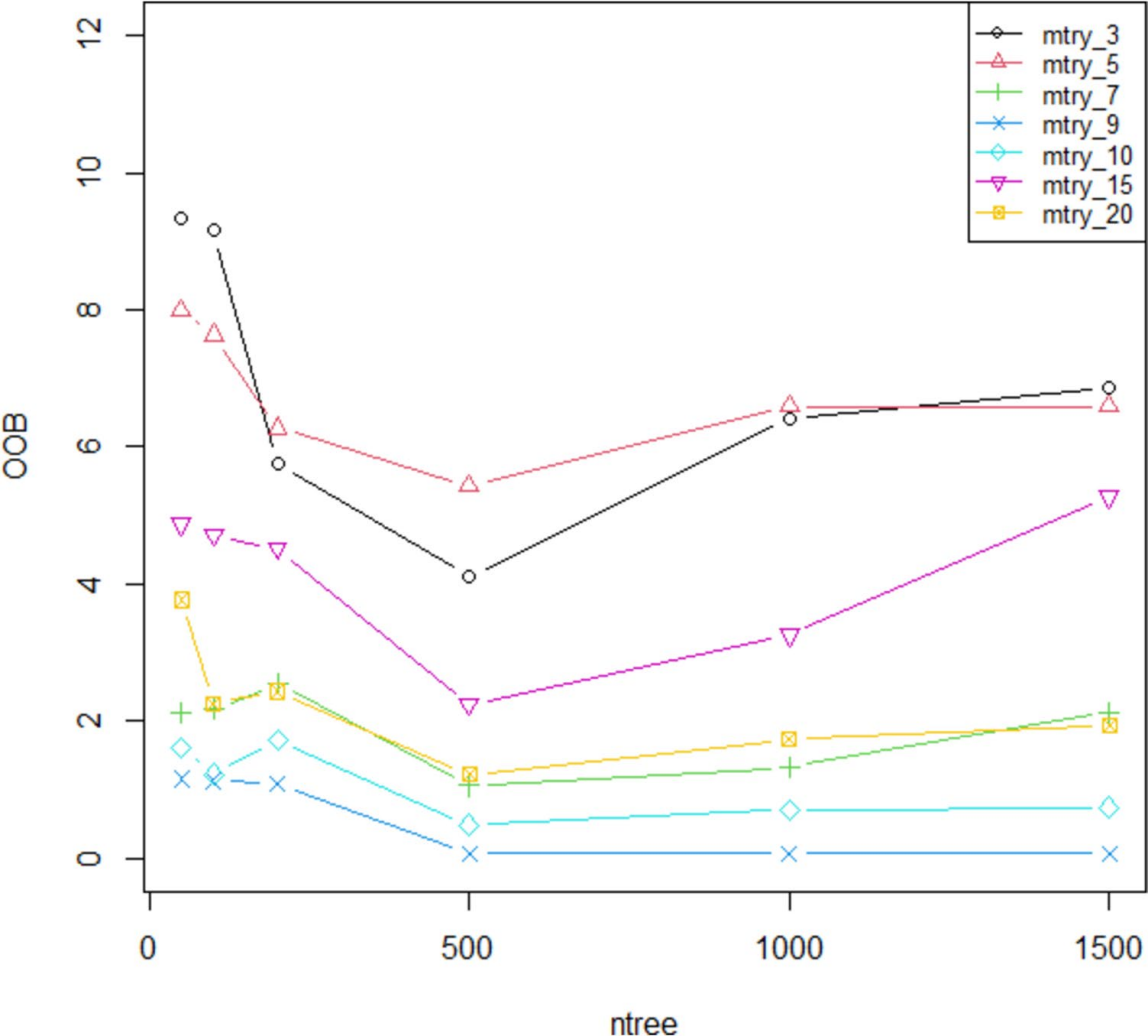
Random forest tuning parameters

Figure 5 presents the out-of-bag (OOB) error rates for random forest model, evaluated across different values of the number of trees (ntree) and the number of features considered at each split (mtry). The OOB error rates reflect the model’s performance in terms of its generalization error during training.

#### Influence of ntree on OOB Error Rate

- Low ntree values (50 and 100): The OOB error rates are relatively high across all mtry values, indicating less stable model performance. For instance, with ntree of 50, the error rates range from 1.16 to 9.34, and with ntree of 100, they range from 1.13 to 9.15. This suggests that a lower number of trees may lead to higher variance in model predictions.
- Intermediate ntree values (200 and 500): As the number of trees increases, the OOB error rates generally decrease. For example, with ntree of 500, the OOB error rates drop significantly, reaching as low as 0.06 for mtry_9 and mtry_10, demonstrating improved model performance and stability with more trees.
- High ntree values (1000 and 1500): The OOB error rates show some variability but generally increase compared to ntree of 500. For instance, with ntree of 1000, the error rates range from 0.06 to 6.59, and with ntree of 1500, the error rates range from 0.06 to 6.87. This increase may suggest a diminishing return on model improvement as the number of trees continues to grow, possibly due to overfitting or increased computational complexity.

#### Influence of mtry on OOB Error Rate

- Low mtry values (3 and 5): Smaller values of mtry tend to result in higher OOB error rates for most ntree values. For example, with ntree of 50, the error rate for mtry_3 is 9.34, which is higher compared to larger mtry values. This indicates that using fewer features at each split may limit the model’s ability to capture complex patterns.
- Optimal mtry values (7, 9, and 10): Moderate mtry values often yield the lowest OOB error rates. For instance, with ntree of 500, mtry_9 and mtry_10 result in error rates of 0.06 and 0.49, respectively, showing improved performance. This suggests that an optimal number of features considered at each split helps in reducing model error.
- High mtry values (15 and 20): Increasing mtry beyond a certain point generally shows higher error rates for lower ntree values but performs better as ntree increases. For example, mtry_15 and mtry_20 exhibit higher error rates compared to moderate mtry values with ntree of 50 and 100 but become more competitive at higher ntree values like 500 and 1000.

### Feature Importance and Variable Selection

**Fig. 6:**
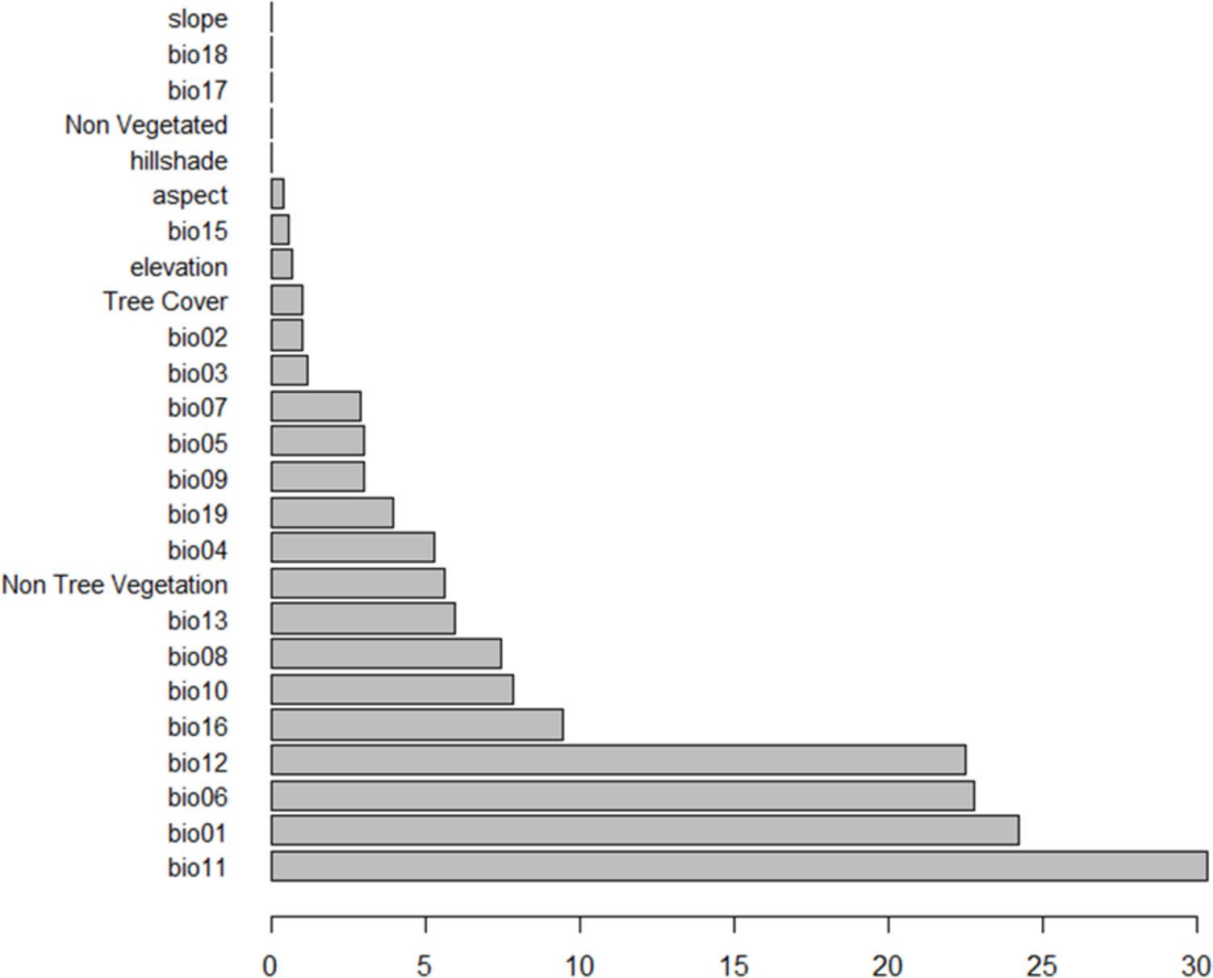
Feature importance as calculated in the trained random forest models

Random forest offers robust methods for evaluating feature importance, aiding in model simplification, and enhancing interpretability. By leveraging these insights, practitioners can build more effective and understandable models while gaining a deeper understanding of the data driving their predictions. Figure 5 and Table 2 reveal five parameters were non-significantly important and would be removed from the random forest model as their importance is zero over all the trained models for the different seeds. These are slope, bio18, bio17, non-vegetated, and hillshade as illustrated in Fig. 6.

**Table 2:** Feature importance for each variable category

a. Temperature-related parameters															
Name	bio1	bio2	bio3		bio4	bio5	bio6			bio7	bio8		bio9	bio10	bio11
Importance	24.2	1.03	1.16		5.31	3.04	22.75			2.92	7.4		3.04	7.86	30.3
b. Precipitation-related parameters															
Name	bio12	bio13	bio15		bio16	bio17	bio18			Bio19					
Importance	22.5	5.96	0.57		9.44	0	0			3.98					
c. Topography-related parameters															
Name	Elevation		Slope			Aspect		Hillshade							
Importance	0.7		0			0.39		0.02							
d. Vegetation-related parameters															
Name	Percent tree cover			Percent non-vegetated					Percent non-tree vegetation						
Importance	1			0					5.65

#### Temperature-Related Parameters

- bio1 (mean annual temperature) and bio11 (mean temperature of the warmest month) are the most influential temperature-related parameters with importance scores of 24.2 and 30.3, respectively. These features contribute significantly to the model’s predictive accuracy.
- bio6 (mean temperature of the coldest quarter) also shows substantial importance (22.75), highlighting its relevance in the model.
- Other temperature-related parameters such as bio4 (temperature seasonality) and bio10 (mean temperature of the coldest month) have moderate importance scores (5.31 and 7.86, respectively), while features like bio2 (mean diurnal range) and bio7 (temperature annual range) contribute less to the model.

#### Precipitation-Related Parameters

- bio12 (annual precipitation) stands out with the highest importance score of 22.5, indicating its significant role in the model.
- bio16 (precipitation of the wettest month) also contributes notably with an importance score of 9.44.
- bio13 (precipitation of the driest month) and bio19 (precipitation of the warmest quarter) have moderate importance (5.96 and 3.98, respectively).
- Other precipitation-related parameters such as bio15 (precipitation seasonality) and bio17 (precipitation of the coldest quarter) are less influential, with scores of 0.57 and 0.

#### Topography-Related Parameters

- Elevation is the most influential topographic parameter with an importance score of 0.7, albeit relatively low compared to temperature and precipitation parameters.
- Aspect contributes moderately with a score of 0.39, while hillshade has minimal importance (0.02).
- Slope is deemed insignificant for this model with an importance score of 0.

#### Vegetation-Related Parameters

- Percent non-tree vegetation holds the highest importance among vegetation-related parameters with a score of 5.65, indicating its relevance to the model.
- Percent tree cover contributes minimally with a score of 1.
- Percent non-vegetated is deemed non-contributory for this model with an importance score of 0.

### Accuracy Assessment

**Fig. 7:**
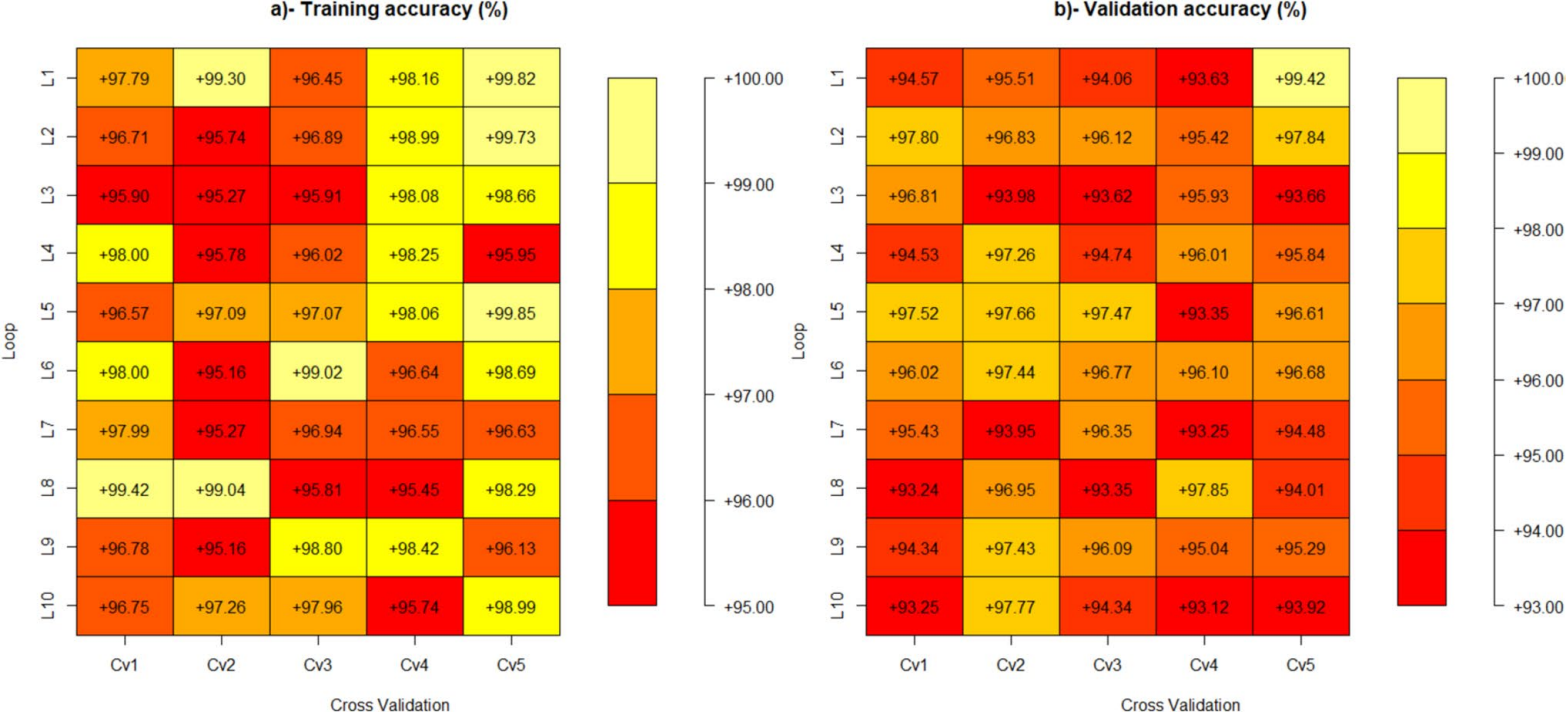
Training and validation accuracy of the tested random forest model (ntree = 500, mtry = 9)

In Fig. 7, we present the performance metrics of the random forest (RF) model following the application of fine-tuned hyperparameters. Specifically, the model was optimized with mtry = 9 and ntree} = 500. The depicted accuracies illustrate the model’s performance across different training and validation loops within each cross-validation fold.

The average training accuracy achieved was 97.34%, reflecting the model’s effectiveness in learning from the training data. Concurrently, the validation accuracy averaged 95.57%, indicating the model’s strong generalization capability to unseen data. These results underscore the robustness of the RF model with the specified hyperparameter settings.

### Habitat Suitability Index

The Habitat Suitability Index (HSI) is an indicator of a habitat’s ability to sustain a particular species. The higher the value is the more suitable the area for the given species where values below 0.6 is considered boor and values higher than this value ranges between average to excellent condition (Oldham et al. 2000). Figure 5 illustrates the spatial distribution of HSI and its values while Fig. 6 quantifies areas per each class in km.^2^

**Fig. 8:**
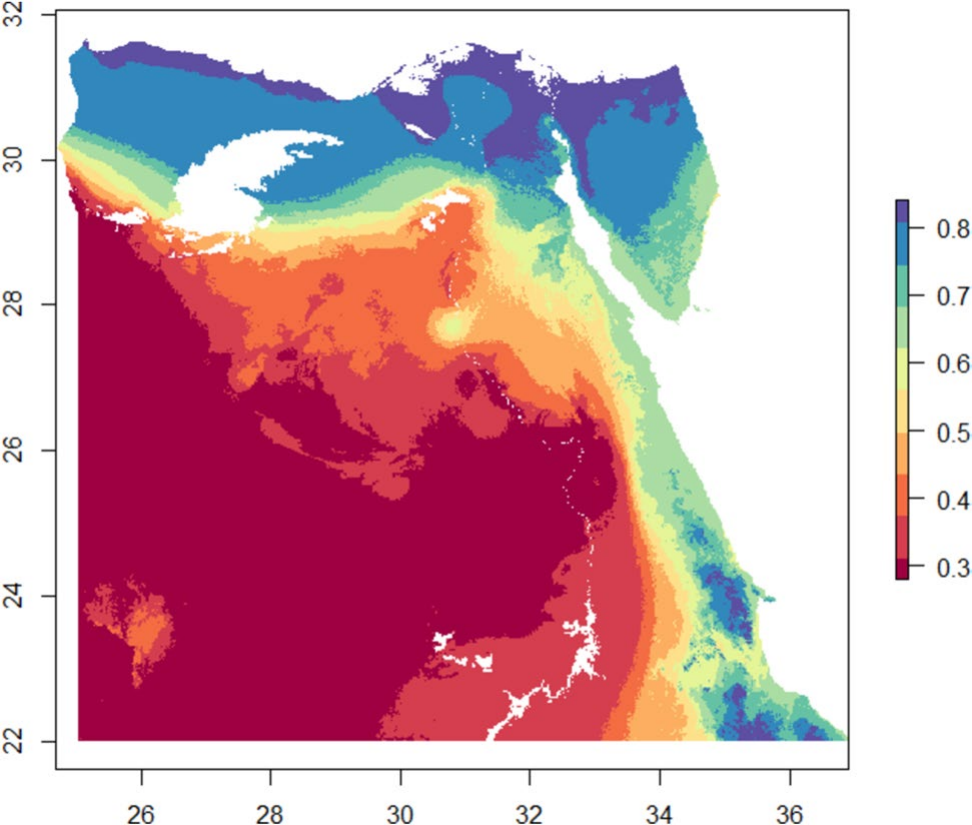
Habitat Suitability Index for *Apis florea* over the study area

From Fig. 8, it is evident that most of the suitable areas are in the eastern and northern parts of Egypt where water and natural resources exist. This also includes the Nile Delta which is characterised by its agriculture lands and the existence of the river Nile, while the western south parts are very poor in terms of suitability index where there is no any green vegetation and low relative humidity (El-Shirbeny and Abdel latif 2017).

**Fig. 9:**
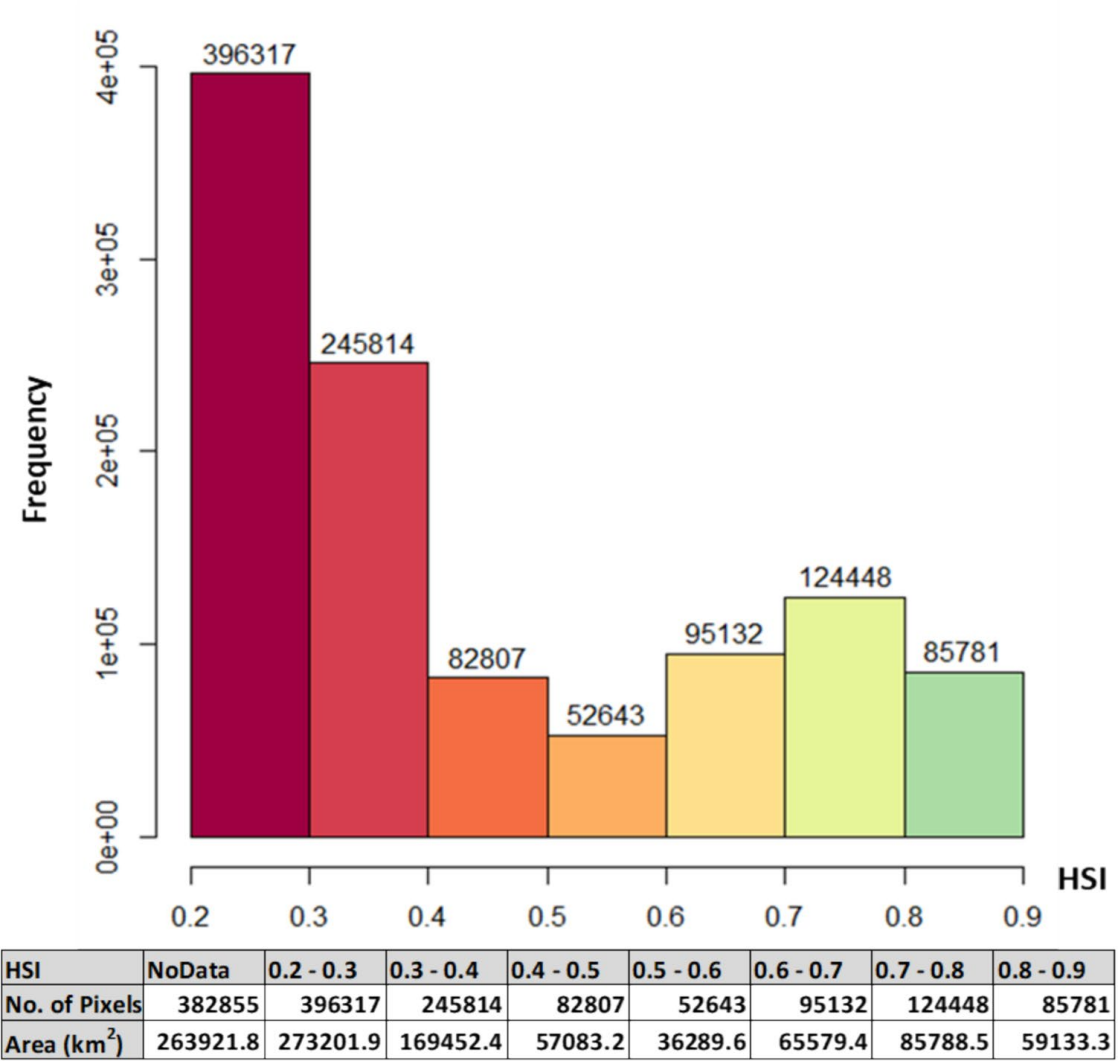
Areas (km^2^) for different HSI classes

According to data illustrated in Fig. 9, it is evident that the area of the habitats varies across the different HSI values. The largest habitat area is observed at HSI ranged between 0.2 and 0.3 with 259744 km^2^, while the smallest habitat area is observed at HSI ranged between 0.5 and 0.6 with 34501.9 km^2^. The total suitable area that has HSI values range between 0.6 and 0.9 is 200131.9 km^2^ of the study area, while the rest areas are not suitable for the *A. florea* in terms of the selected variables (Oldham et al. 2000).

This finding aligns with the concept of habitat fragmentation, where habitats with higher HSI values tend to be smaller and more isolated. Fragmentation can have detrimental effects on biodiversity by reducing gene flow, increasing edge effects, and decreasing habitat connectivity (Fahrig 2003; Haddad et al. 2015).

Further research is needed to investigate the specific factors influencing habitat stability and their effects on species richness and population dynamics. Incorporating temporal data and analysing trends over time would provide valuable insights into the long-term changes in habitat stability and the associated implications for biodiversity conservation.

**Fig. 10:**
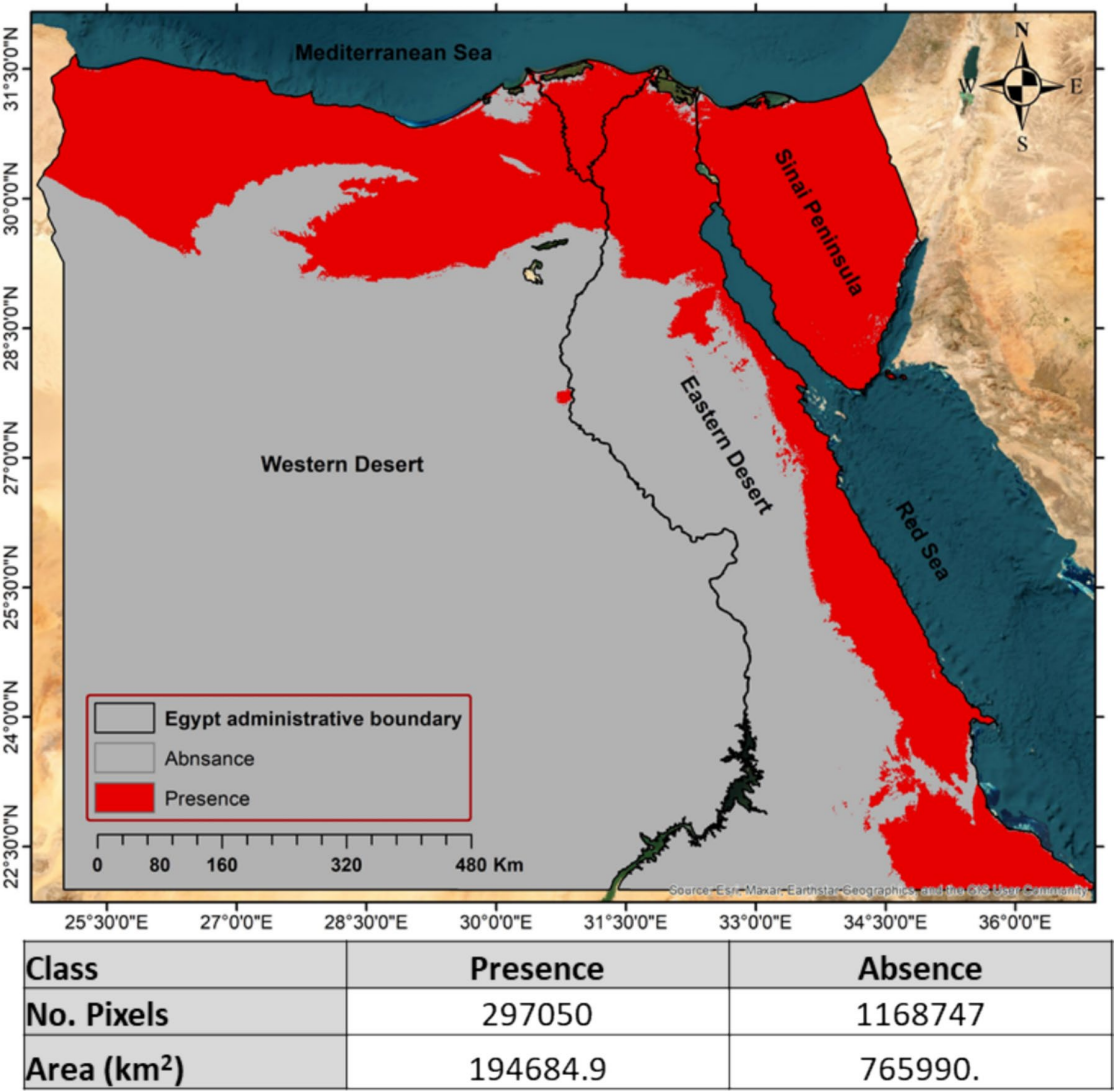
Potential distribution

Figure 10 illustrates the final random forest predicted area of presence of *Apis florea*. The total area of absence is 802322.9 km^2^ (~ 79.4%) while the area of presence is 208127.1 km^2^ (~ 20.6%).

## Discussion

The environmental parameters of the study area in Egypt indicate a habitat suitable for *A. florea*, with diverse topographic features and a range of climatic conditions. The area experiences high temperatures during the warmest quarter, with a mean temperature of 16.4 to 34.2°C, while the coldest quarter has a moderate to low temperatures (1.5 to 21.6°C). Annual precipitation is low, with a median of 6mm, and the wettest month receives only 2mm of precipitation. There is little variation in precipitation between seasons, and the driest quarter has relatively low rainfall (62mm). The elevation ranges from 1 to 2467m, providing varied habitats, and the slope varies from 1 to 25°. The aspect and hillshade values indicate diverse exposure to sunlight and wind. The percentage of tree cover ranges from 1 to 38, while non-tree vegetation covers 1 to 87% of the area. However, there are also non-vegetated areas, ranging from 4 to 100%. These environmental factors contribute to the suitability of the habitat for *A. florea* in Egypt (Beaumont et al. 2005; Elith et al. 2006; Abutaleb et al. 2019; Parichehreh et al. 2022; Silva et al. 2020).

In summary, the analysis demonstrates that temperature-related parameters, especially bio1, bio6, and bio11, are the most influential features in the random forest model. Precipitation-related parameters also play a significant role, particularly bio12 and bio16. Topographic parameters generally have lower importance, with elevation being the most relevant. Among vegetation-related parameters, percent non-tree vegetation is the most impactful. This detailed breakdown of feature importance helps in understanding which environmental factors most significantly affect the model’s predictions; results agreed with Ruttner and Ruttner (1988) who found that *A. florea* can endure an extremely hot and dry climate and can withstand summer temperatures that reach 50°C and more (Whitcombe 1984; Ruttner et al. 1985) was discovered in a subtropical climate (less than 5 days of temperatures below + 5°C) and was able to survive cold spells during the winter. Whitcombe (1984) discovered that *A. florea* appears to have a better individual temperature tolerance than *A. mellifera* since it persists during the hours when the temperature is above 40°. Hepburn and Radloff (2011) show that *A. florea* are quite adaptive when breeding and appear to find acceptable sites under a wide range of environmental circumstances, and an analysis of their global distribution shows that they are a subtropical species. Also, this result is compatible with Abou-Shaara et al. (2021) who find that highly suitable ranges of these variables for red dwarf bees. These ranges were 21–24°C, 11–13°C, 34–36°C, 7–9°C, 27–29°C, and 14–15°C for bio1, bio2, bio5, bio6, bio10, and bio11, respectively. Thus, the perfect range across all variables was from 7 to 36°C.

Bajiya and Abrol (2017) concluded that *A. florea*’s foraging population was considerable and positively linked with both the warmest and coldest temperatures. Cui and Corlett (2016) also found that the greatest temperature that *A. florea* could tolerate was 46.5°C, and the minimum temperature thresholds for its activity demonstrated a positive connection with its abundance was 16°C.

Bajiya and Abrol (2017) found that the foraging activity of *A. florea* decreased with relative humidity and rainfall. Also, Cui and Corlett (2016) found that *A. florea* sometimes became active in light rain, but heavy rain prevented their all activity. On the other hand, Hepburn and Radloff (2011) demonstrated how *A. florea* is always linked to the following occurrences: rainfall > flowering > swarming or migration.

Lindauer (1957) recorded a brood comb temperature of 34–36°C. Whitcombe (1984) claims that the quantity of shade, which in open-air nests in Oman was found to average 75%, is a crucial element in preserving the microclimate of the nest. This level of shadowing appears to represent a compromise between the colony’s needs for microclimate, predator protection, and orientation; it is thought that the colony will eventually move in order to achieve balance. Because it offers a greater temperature in the morning and shade in the afternoon, the SE sector of a tree is undoubtedly favoured as a nesting place.

Shebl (2017) discovered that *Ficus nitida* trees, followed by guava, mango, palm, and camphor trees at a high height, are where dwarf honey bees like to establish their nests. Maa (1953) and Free (1981) found that Pakistan, India, Sri Lanka, Thailand, Indochina, Malaysia, sections of Indonesia (Sumatra, Java, Borneo), and Palawan (but not the other islands of the Philippines) are the species’ principal habitats, and colonies have been seen up to 900m in Iran (Ruttner et al. 1985) and up to 1900m in Oman (Whitcombe 1984).

Result strongly agreed with the finding of Shebl (2017), who revealed that a number of colonies were present in the area, nesting in trees along the Suez Gulf, including *Ficus nitida*, *Eucalyptus* species, *Guava* species, *Mangifera indica*, and palm trees. Although there are no agricultural crops in the area, *Ocimum basilicum*, sunflower, citrus, and *Lantana camara* blooming species do exist. On the other hand, results are incompatible with the finding of Free (1981) and Booncham et al. (1995), as they stated that compared to *A. andreniformis*, they claimed that *A. florea* nests are frequently discovered in disturbed regions and can be found in both urban and agriculturally active areas as well as savanna.

The analysis indicates that an increased number of trees (ntree) generally improves model performance, as evidenced by reduced OOB error rates up to a certain point. The mtry parameter also plays a crucial role, with moderate values often providing the best balance between model complexity and accuracy. Optimal tuning of both ntree and mtry is essential for minimizing OOB error and achieving robust model performance.

The variation in habitat areas observed in this study can be attributed to several factors. Habitat stability is influenced by ecological processes such as climate, topography, vegetation cover, and human activities. The results of this study can aid in the identification and prioritization of areas with high habitat stability for conservation measures (Fahrig 2003; Haddad et al. 2015).

## Conclusions

The presence of *Apis florea* has been recently confirmed in Egypt, marking the second documented occurrence of this species on the African continent. This study aimed to comprehensively investigate the distribution of *A. florea* in Egypt and assess its potential for invasive behaviour. Over a span of 2 years, extensive field surveys were conducted, yielding valuable spatial data on the distribution of these red dwarf honeybees.

The study employed an exhaustive analysis approach, utilizing long-term monthly temperature and rainfall data to generate high-resolution spatial climate surfaces with a 1-km resolution. Furthermore, vegetation variables derived from Terra MODIS data were integrated into the analysis. Additionally, elevation data from the Shuttle Radar Topography Mission were utilized to derive slope, aspect, and hillshade characteristics based on digital elevation models. Data underwent resampling to achieve optimal smoothing.

Subsequently, a random forest model was applied to the processed data, followed by a meticulous accuracy assessment to rigorously evaluate the classification output. The results pinpointed that temperature-related parameters, especially bio1, bio6, and bio11, with precipitation-related parameters particularly bio12 and bio16 as key determinants of habitat suitability for *A. florea*. Topographic parameters generally have lower importance. Vegetation-related parameter, percent non-tree vegetation, is the most impactful.

The analysis revealed that the most suitable habitats, covering 200131.9 km^2^, were predominantly situated in Egypt’s eastern and northern regions. These areas include the fertile agricultural lands of the Nile Valley and Delta and benefit from the presence of the Nile River, providing a consistent water source. In contrast, the western and southern parts exhibited low habitat suitability due to limited green vegetation and low relative humidity.

In conclusion, this study provides valuable insights into the distribution and habitat suitability of *A. florea* in Egypt. It has pointed to the possibility of *A. florea* settlement in the Nile Valley and Delta region. The study indicates the necessity of conducting more studies to evaluate the risks of further distribution of *A. florea* in Egypt as it can affect the conservation of the local species of pollinating insects, especially local honey bee strains.

## Data Availability

The datasets generated during and/or analysed during the current study are available from the corresponding author on reasonable request.

## Code Availability

Not applicable (Google Earth Engine and random forest-Free).
